# Hypothesis and Holistic Knowledge of Testicular Torsion Among the General Population of the Kingdom of Saudi Arabia

**DOI:** 10.7759/cureus.51194

**Published:** 2023-12-27

**Authors:** Waleed K Alghuyaythat, Mohammad Alkhamees, Abdulaziz S Alanazi, Khalid F Alotaibi, Ahmed M Kaseb, Abdulaziz M Alanazi, Murad M Almutairi, Faisal K Alhusini, Abdulrahman N Almutairi, Bassam M Aldhafeeri, Abdullah O Aldahash, Saad R Aljameely, Mohammad O Alenazi

**Affiliations:** 1 College of Medicine, Majmaah University, Al-Majmaah, SAU; 2 Department of Urology, College of Medicine, Majmaah University, Al-Majmaah, SAU; 3 Department of Medicine, Majmaah University, Al-Majmaah, SAU; 4 Department of Family Medicine, King Khalid General Hospital, Hafr Al-Batin, SAU; 5 Department of Ear, Nose, and Throat, Northern Border University, Arar, SAU

**Keywords:** urology department, knowledge, urology, saudi arabia, emergency, testicular torsion

## Abstract

Background

Testicular torsion is a serious condition that requires immediate medical attention. It occurs when the spermatic cord, which carries blood to the testicles, twists, reducing blood flow and oxygen to the testicle. This can lead to tissue death and loss of the testis if not treated promptly. It is important to seek medical attention immediately when symptoms of testicular torsion arise, as prompt treatment can help prevent permanent damage to the testicle. This study aimed to explore the level of knowledge about testicular torsion among the general population in the Kingdom of Saudi Arabia.

Methodology

A descriptive cross-sectional study was conducted using a convenience sample recruited from the general population who fulfilled the inclusion criteria. The data were collected from participants using an electronic pre-structured questionnaire. The researchers developed the questionnaire through expert consultation and after an intensive literature review. The questionnaire was reviewed by a panel of three experts for validation and applicability. After a pilot study, the reliability coefficient (Cronbach’s alpha) was 0.74. The data were analyzed using SSPS version 25 (IBM Corp., Armonk, NY, USA) and presented as percentages and frequencies. Chi-square and logistic regression were conducted. P-values ≤0.05 were considered statistically significant.

Results

A total of 732 participants were recruited, most of whom were male (486, 66.4%), with ages ranging between 18 and 30 years (452, 61.7%). Regarding testicular torsion knowledge, more than half of the participants had good knowledge (406, 55.5%) and knew about the signs, symptoms, and risk factors of testicular torsion. There was a statistically significant relationship between age and knowledge (p < 0.001) and an insignificant relationship between sex and knowledge (p > 0.05).

Conclusions

The study participants were found to have good knowledge. Fortunately, most participants knew that testicular torsion is an emergency and they must immediately visit the hospital. With further awareness programs, the overall knowledge level can be improved.

## Introduction

Testicular torsion is a common urological emergency that involves the rotation of the testis and the twisting of the spermatic cord, resulting in a reduced blood supply to the affected testis, which causes atrophy of the testis. The testis is particularly prone to ischemic injury because the blood supply is terminal (i.e., the arteries in the testes do not form anastomoses) and because the tunica albuginea is an inelastic shell that restricts compensatory testis expansion during trauma [1,2].

Mostly, newborns, kids, and teenagers are affected by testicular torsion. Testicular torsion has two distinct forms, namely, intravaginal torsion, which more frequently occurs throughout adolescence, and supravaginal torsion, which occurs during puberty and the first year after birth [3]. Trauma-induced testicular torsion is a rare condition that often takes time to be diagnosed, which can result in a poor rate of testicular salvation. Emergency surgeons, particularly those working in nearby hospitals, should be aware of the potential for testicular torsion in children who have experienced testicular trauma. Increasing parental knowledge about testicular torsion is also crucial [4].

A surgical procedure is required to establish the counter-rotation of the twisted spermatic cord and reperfusion of the tissue. One in 4,000 males under the age of 25 and one in 160 males above the age of 25 are affected by testicular torsion, accounting for 13-54% of cases of acute pediatric scrotal disease [5-7]. If the spermatic cord is detached within six hours after the start of torsion, there is a 90% chance that the testicles will survive. However, this rate drops to 50% after 12 hours and to 10% after 24 hours [8]. Testicular injury caused by torsion cuts off blood flow to the testis and surrounding structures in the scrotum, and subsequent detorsion, which restores blood flow, results in altered hormone production, subfertility, and infertility, leading to a burst of oxygen-derived free radicals that cause additional injuries to the testis. The main pathophysiological effect of testicular torsion is ischemia damage, resulting from the twisting of the spermatic cord, which causes the tissue to become ischemic and then reperfusion upon release [9,10].

The length of torsion and the degree of twisting of the spermatic cord are the two main factors that aggravate the severity of testicular injury. Infertility may develop from ischemia-reperfusion injury, which causes neutrophil recruitment, the production of reactive oxygen species, the release of proinflammatory cytokines and adhesion molecules, lipid peroxidation, apoptosis, anoxia, and changes to microvascular blood flow [11,12].

This study aims to explore the level of knowledge about testicular torsion among the general population in the Kingdom of Saudi Arabia, identify the hypothesis regarding testicular torsion, and determine the level of knowledge regarding testicular torsion among the participants.

## Materials and methods

Study design and population

An observational, descriptive, cross-sectional, community-based study was conducted among the general population in the Kingdom of Saudi Arabia to evaluate the awareness of testicular torsion.

Participants

Adult (18 years old and above) Saudi Arabians of both genders who showed a willingness to participate in the study were included. Individuals less than 18 years old who did not show a willingness to participate were excluded. A convenience sampling technique was used in the study to collect the responses from the Saudi Arabian general population.

The sample size was determined using the equation n= z^2^ × p × q/d^2^ as n = (1.96)^2^ × 0.5 × 0.5 ÷ (0.05)^2^ = 385, where n refers to sample size, z is the standard deviation (1.96), p is the proportion of population (0.5), d is the degree of precision (0.05), and q is the error sample (1 -p).

The minimum sample size required was 385 participants. The final sample size of our study was 732 participants.

Data collection tool

The tool was developed by the researchers with the guidance of the previous literature. The first part of the questionnaire included questions about the demographic data, such as sex and age. The second part included questions about the knowledge of participants about testicular torsion knowledge and awareness. It also included questions on knowledge about causes, etiology, signs, symptoms, risk factors, and management of testicular torsion. The questions were developed in the form of multiple-choice questions, and the participants were asked to respond by selecting the right answer.

The questionnaire was developed in Arabic and the data were collected in Arabic as multiple-choice questions, which were then translated back to English. The data collection tool was validated by a panel of three expert consultant urologists to ensure its content validity and applicability. The reliability of the questionnaire was assessed through a pilot study involving 35 participants, resulting in a reliability coefficient (Cronbach’s alpha) of 0.74. Participants in the pilot study were excluded from this study.

Data analysis

The data were analyzed using the statistical SPSS version 25 (IBM Corp., Armonk, NY, USA). Descriptive statistics were applied for percentages and frequencies. Associations between variables were identified using logistic regression to identify if any one of the general characteristics of the participants was a predictor for good or poor knowledge. The chi-square test was conducted to identify statistical significance. P-values ≤0.05 were considered statistically significant.

Ethical considerations

Ethical approval was obtained from the Majmaah University Research Ethics Committee (approval number: MUREC-DEC.3/COM-2023/34-3). The participants were informed about the research, and informed consent was obtained before filling out the questionnaire. The responses were kept completely confidential and used only for research purposes.

## Results

Of the 732 study participants, 486 (66.4%) were male and 246 (33.6%) were female. The majority were aged 18-30 years (452, 61.7%), followed by 21-45 years (162, 22.1), 46-60 years (85, 11.6%), and more than 60 years (33, 4.5%). A little more than half had good knowledge regarding testicular torsion (406, 55.5%), and the rest had poor knowledge (326, 44.5%). There was a significant relationship between age and knowledge (p < 0.001) (Table 1).

**Table 1:** Bivariate analysis of general characteristics, knowledge questions, and testicular torsion knowledge among the general population of the Kingdom of Saudi Arabia (N = 732).

Parameter		Total, n (%)	Testicular torsion knowledge, n (%)		P-value
			Good; 406 (55.5)	Poor; 326 (44.5)	
Age	18–30 years	452 (61.7)	280 (38.3)	172 (23.5)	<0.001
	31–45 years	162 (22.1)	74 (10.1)	88 (12.0)	
	46–60 years	85 (11.6)	36 (4.9)	49 (6.7)	
	More than 60 years	33 (4.5)	16 (2.2)	17 (2.3)	
Sex	Male	486 (66.4)	271 (37.0)	215 (29.4)	0.441
	Female	246 (33.6)	135 (18.4)	111 (15.2)	
Did you know what testicular torsion is?	Yes	269 (36.7)	190 (26.0)	79 (10.8)	<0.001
	No	272 (37.2)	114 (15.6)	158 (21.6)	
	I think I heard about it, but I don’t know	191 (26.1)	102 (13.9)	89 (12.2)	
Is testicular torsion a pathogenic disease?	Viral disease	138 (18.9)	45 (6.1)	93 (12.7)	<0.001
	Bacterial disease	81 (11.1)	28 (3.8)	53 (7.2)	
	No	307 (41.9)	242 (33.1)	65 (8.9)	
	I don’t know	206 (28.1)	91 (12.4)	115 (15.7)	
Is testicular torsion an emergency?	Yes	555 (75.8)	362 (49.5)	193 (26.4)	<0.001
	No	177 (24.2)	44 (6.0)	133 (18.2)	
If the patient suspects testicular torsion, when should they go to the hospital?	Immediately	453 (61.9)	330 (45.1)	123 (16.8)	<0.001
	Free time	118 (16.1)	27 (3.7)	91 (12.4)	
	Wait until booking an appointment with the urologist	116 (15.8)	37 (5.1)	79 (10.8)	
	Stay at home and take analgesics only	45 (6.1)	12 (1.6)	33 (4.5)	
Is testicular torsion a painless condition?	Yes	204 (27.9)	94 (12.8)	110 (15.0)	<0.001
	No	234 (32.0)	172 (23.5)	62 (8.5)	
	I don’t know	294 (40.2)	140 (19.1)	154 (21.0)	
If the patient feels severe pain within the scrotum, what should they do?	Go immediately to the emergency room	449 (61.3)	328 (44.8)	121 (16.5)	<0.001
	Rest and avoid movement	115 (15.7)	30 (4.1)	85 (11.6)	
	Be patient until the pain resolves	70 (9.6)	18 (2.5)	52 (7.1)	
	I don’t know	98 (13.4)	30 (4.1)	68 (9.3)	
Most cases of testicular torsion are idiopathic	Yes	443 (60.5)	300 (41.0)	143 (19.5)	<0.001
	No	289 (39.5)	106 (14.5)	183 (25.0)	
Testicular torsion starts as	Mild symptoms	67 (9.2)	15 (2.0)	52 (7.1)	<0.001
	Gradient symptoms	244 (33.3)	120 (16.4)	124 (16.9)	
	Intermittent symptoms	113 (15.4)	38 (5.2)	75 (10.2)	
	Severe symptoms	308 (42.1)	233 (31.8)	75 (10.2)	
In which period does the risk increase to developing testicular torsion?	10–20 years	185 (25.3)	91 (12.4)	94 (12.8)	0.042
	21–30 years	194 (26.5)	106 (14.5)	88 (12.0)	
	More than 30 years	192 (26.2)	106 (14.5)	86 (11.7)	
	The risk doesn’t increase with a certain period	161 (22.0)	103 (14.1)	68 (7.9)	
Can testicular torsion be treated by analgesia, antibiotics, and observation only?	Yes	216 (29.5)	89 (12.2)	127 (17.3)	<0.001
	No	257 (35.1)	201 (27.5)	56 (7.7)	
	I don’t know	259 (35.4)	116 (15.8)	143 (19.5)	
In case of prolonged duration of testicular torsion and signs of death, how should the doctor deal with it?	Remove it	292 (39.9)	117 (24.2)	115 (15.7)	0.018
	Keep it in place	126 (17.2)	76 (10.4)	50 (6.8)	
	Examine it only without any surgical intervention	89 (12.2)	43 (5.9)	46 (6.3)	
	I don’t know	225 (30.7)	110 (15.0)	115 (15.7)	
Does vigorous exercise protect from testicular torsion?	Yes	218 (29.8)	92 (12.6)	126 (17.2)	<0.001
	No	238 (32.5)	180 (24.6)	58 (7.9)	
	I don’t know	276 (37.7)	134 (18.3)	142 (19.4)	
One of the etiology of testicular torsion is	Congenital causes	161 (22.0)	63 (8.6)	98 (13.4)	<0.001
	A little movement	104 (14.2)	39 (5.3)	65 (8.9)	
	Sitting for a long time	58 (7.9)	24 (3.3)	34 (4.6)	
	All of the above	409 (55.9)	280 (38.3)	129 (17.6)	

Regarding knowledge about testicular torsion among participants, when asked if they knew what testicular torsion is, the majority said no (272, 37.2%), followed by yes (269, 26.7%), and 191 (26.1%) said that they had heard about it but they did not know. Regarding testicular torsion as a pathogenic disease, the majority said no (307, 41.9%), followed by do not know (206, 28.1%), viral disease (138, 18.9%), and bacterial disease (81, 11.1%). Regarding testicular torsion as an emergency, most participants said yes (555, 75.8%), and 177 (24.2%) said no. When asked if a patient suspects testicular torsion, when should they go to the hospital, most said immediately (453, 61.9%), followed by in their free time (118, 16.1%), waiting until they book an appointment with the urologist (116, 15.8%), and 45 (6.1%) said to stay at home and take analgesics (Table 1).

Regarding testicular torsion being a painless condition, the majority said they do not know (294, 40.2%), followed by no (234, 32.0%), and 204 (27.9%) said yes. When asked if a patient feels severe pain within the scrotum, what should they do, most participants (449, 61.3%) said to go immediately to the emergency room, followed by rest and avoiding movement (115, 15.7%), do not know (98, 13.4%), and 70 (9.6%) said to be patient until the pain resolves. When asked if most cases of testicular torsion are idiopathic, the majority said yes (443, 60.5%), and 289 (39.5) said no (Table 1).

When asked about the onset of the testicular torsion symptoms, the majority said severe symptoms (308, 42.1%), followed by gradient symptoms (224, 33.3%), intermittent symptoms (113, 15.4%), and mild symptoms (67, 9.2%). Regarding at which period the risk increases to testicular torsion, the participants answered approximately the same with 194 (26.5%) responding 21-30 years, 192 (26.2%) more than 30 years, 185 (25.3%) 10-20 years, and 161 (22.0%) responding that the risk did not increase with a certain period. When asked if testicular torsion can be treated with analgesia, antibiotics, and observation, the majority said they did not know (259, 35.4%), followed by no (257, 35.1%), and yes (216, 29.5%) (Table 1).

When asked about cases of prolonged duration of testicular torsion and signs of death, how the doctor should deal with the testis, the majority said to remove it (292, 39.9%), followed by do not know (225, 30.7%), keep it in place (126, 17.2%), and examine it only without any surgical intervention (89, 12.2%). When asked if vigorous exercise protects from testicular torsion, the majority said they do not know (276, 37.7%), followed by no (238, 32.5%), and yes (218, 29.8%). About the etiology of testicular torsion, 161 (22.0%) said congenital causes, 104 (14.2%) little movement, 58 (7.9%) sitting for a long time, and most (409, 55.9%) reported all of these causes (Table 1).

Pearson’s chi-square test of significance was conducted between the knowledge questions and testicular torsion knowledge categories (good or poor). All questions showed a significant relationship with p-values of less than 0.05, with most showing p-values of less than 0.001. The frequencies and percentages for each question with testicular torsion knowledge categories are shown in Table 1.

As shown in Table 2, the logistic regression estimates for all participants showed no important effect regarding age group or sex on testicular torsion knowledge. The odds ratio (OR) among age groups was 1.947 for the 31-45 years group, 2.228 for the 46-60 years group, and 1.729 for the more than 60 years group. The p-values of the Wald test were insignificant among the more than 60 years age group (0.130). Significant results were noted for the 31-45 years and 46-60 years age groups (0.001 and 0.001, respectively). Hence, the possibility of having good knowledge among these groups was less than the possibility of having poor knowledge. The OR among the male group was 0.921 with an insignificant p-value of the Wald test (0.605) The value of Nagelkerke was 0.038. This meant the interpreted variation explained by the model (3.8%) was a very small value.

**Table 2:** Logistic regression estimates of age and sex based on testicular torsion knowledge.

Parameter		B	Significance	Odds ratio	95% confidence interval for odds ratio	
					Lower bound	Upper bound
Age	18–30 years	Reference	0.000	-	-	-
	31–45 years	0.666	0.001	1.947	1.354	2.801
	46–60 years	0.801	0.001	2.228	1.391	3.568
	More than 60 years	0.548	0.130	1.729	0.851	3.513
Sex	Male	-0.083	0.605	0.921	0.673	1.260
	Female	Reference	-	-	-	-
Constant		-0.434	0.002	0.648	-	-

## Discussion

Testicular torsion refers to the rotation of the testis and the twisting of the spermatic cord, which is a common urological emergency. In this observational, descriptive, cross-sectional study, we collected data from 732 participants from the general population of Saudi Arabia to evaluate their awareness of testicular torsion. Most participants were male (486, 66.4%), and the remaining were female (246, 33.6%). We included females because their children, husbands, or brothers can face this medical emergency, and their knowledge about this condition can save their testis. Regarding the age of the participants, most were between 18 and 30 years old (452, 61.7%), and the remaining were between 31 and more than 60 years old.

Regarding testicular torsion knowledge, 406 (55.5%) participants had good knowledge, and the remaining 326 (44.5%) had poor knowledge. Although the number of participants with good knowledge was higher, the high percentage of participants with poor knowledge is concerning because this emergency condition requires prompt action to save testis and achieve better outcomes.

Among all participants, 555 (75.8%) said testicular torsion is a medical emergency condition, and 453 (61.9%) said that the patient must go to the hospital immediately if they suspect testicular torsion. This is different from a study that included 200 parents (100 parents from two clinics), where 85% and 82% said they would take their kids to the hospital after work if they were experiencing scrotal pain during business hours [13].

Regarding testicular torsion being a painless or painful condition, the majority said they do not know (294, 40.2%), and 308 (42.1%) said that testicular torsion starts with severe symptoms. When asked if a patient feels severe pain within the scrotum, what should they do, most (449, 61.3%) said that they should immediately go to the emergency room, and the remaining said to rest and avoid movement, wait for the pain to resolve by itself, or do not know. In a previous study, 29 (30%) children went to the hospital within six hours of the onset of the pain, and only 17 children (22%) went to the emergency room. Another study found that only seven (22.6%) patients presented to the hospital on time. The difference in findings could be due to the comparable studies presenting the actions of the participants whereas our study asked about what they must do if they had testicular torsion [14,15]. Another study found that parents with high knowledge were four times more likely to arrive at the right time, which is similar to our study that found a significant relationship between going immediately to the emergency room with testicular torsion knowledge (p < 0.001) [16].

Regarding etiological factors and risk factors, most said that the cause is idiopathic (443, 60.5%). Moreover, the majority of participants (409, 55.9%) identified congenital causes, little movement, and sitting for a long time as etiological factors. When asked if vigorous exercise is a protective or risk factor, 238 (32.5%) said risk factor, 218 (29.8%) said protective, and the majority said they do not know (276, 37.7%). Generally, the participants’ knowledge regarding risk and etiological factors was good, which can be improved with further awareness programs.

The major limitation of this study is that because it was conducted through an online survey, the data presented cannot be generalized.

## Conclusions

Among the 732 participants, the majority were male, between 18 and 30 years old, and had good knowledge regarding testicular torsion. The majority identified the etiology, risk factors, signs, and symptoms of testicular torsion. Moreover, most identified testicular torsion as an emergency and stated that they would go to the emergency room immediately if they suspected it. In general, this study addresses important findings regarding the knowledge of the participants. With educational programs about emergency cases and testicular torsion, the knowledge can be further improved. Moreover, there is a need to increase awareness among parents, teachers, coaches, and those in the athletic fields regarding the critical importance of timely presentation in such cases. Future research with a larger sample size and interviews with research participants are required.
